# Synthesis and biological evaluation of novel thienopyrimidine derivatives as diacylglycerol acyltransferase 1 (DGAT-1) inhibitors

**DOI:** 10.1080/14756366.2019.1693555

**Published:** 2019-11-22

**Authors:** Dong Jin Hong, Seung Hyun Jung, Jisook Kim, Danbee Jung, Young Gil Ahn, Kwee Hyun Suh, Kyung Hoon Min

**Affiliations:** a College of Pharmacy, Chung-Ang University, Seoul, Republic of Korea; b Hanmi Research Center, Hanmi Pharm. Co., Ltd., Gyeonggi-Do, Republic of Korea

**Keywords:** DGAT-1, obesity, small molecules, thienopyrimidine, type 2 diabetes

## Abstract

A novel series of thieno[3,2-*d*]pyrimidine derivatives were synthesised and their inhibitory effects against diacylglycerol acyltransferase 1 (DGAT-1) were assessed. cis-Isomer **17a** showed potent and selective inhibitory activity against DGAT-1 in SF9 cells. In addition, **17a** had an acceptable pharmacokinetic profile and accumulated mainly in the small intestine and liver. Oral administration of **17a** led to a significant reduction in plasma triacylglycerol level during an oral lipid tolerance test (OLTT) in murine and canine models. Taken together, **17a** is a high-quality candidate that deserves further investigation.

## Introduction

Obesity is characterised by abnormal fat accumulation due to a systemic dysfunction of energy homeostasis. It is a significant risk factor for diabetes, hypertension, cardiovascular disease, and non-alcoholic fatty liver disease^1^. Excessive accumulation of triglycerides (or triacylglycerol, TAG) in adipocytes and non-adipocytes, including in the liver, skeletal muscle, myocardium, and pancreas, is a crucial feature of obesity^2^. In particular, abnormal TAG levels increases the risk of metabolic syndrome, characterised by as insulin resistance, dyslipidemia and cardiomyopathy^3^.

**Figure 1.**
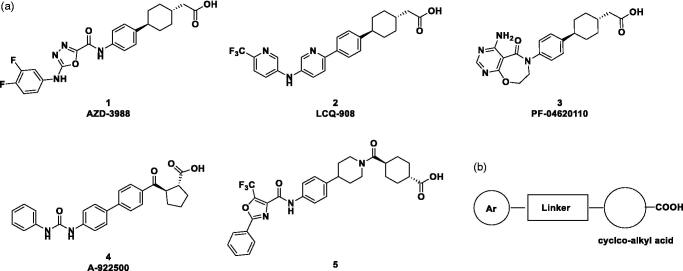
(a) Structures of reported DGAT1 inhibitors; (b) diagram showing structural features of DGAT-1 inhibitors.

Therefore, preventing excessive accumulation of TAG could be beneficial in the treatment of metabolic diseases such as obesity and type 2 diabetes. Two routes of TAG biosynthesis have been identified: the glycerol phosphate pathway, and the monoacylglycerol pathway^4^. Both routes have a common intermediate, diacylglycerol (DAG), which is then converted to TAG by acyl CoA: diacylglycerol acyltransferase (DGAT)^5^. DGAT exists as two isoforms, DGAT-1 and DGAT-2, which share minimal sequence homology^6^. These enzymes catalyse the final dedicated step in TAG synthesis: i.e. the esterification of a fatty acid moiety to DAG to generate TAG. DGAT-1 is highly expressed in the small intestine, adipose tissue, and liver^6^. DGAT-1 deficient mice showed a significant reduction in the postprandial increase of plasma TAG and were resistant to diet-induced obesity due to increased energy expenditure. Moreover, the *DGAT1* knockout mice had enhanced sensitivity to insulin and leptin. Indeed, DGAT-1 inhibitors had significant pharmacologic effects, including decreased TAG, which were consistent with the *DGAT1* knockout mice phenotypes^7^. However, *DGAT2* knockout mice had extremely low levels of TAG, and their skin could not protect against moisture, so these mice died after birth^8^. Therefore, selective DGAT-1 inhibitors have been developed to manage metabolic diseases such as obesity and type 2 diabetes^9–11^. Pharmaceutical companies, including AstraZeneca (**1**)^12^, Novartis (**2**)^13^, Pfizer (**3**)^14^, and Abbott (**4**)^15^, and Hoffmann-La Roche (**5**)^16^ are developing selective DGAT-1 inhibitors, whose structures are described in Figure 1. Other novel DGAT1 inhibitors have also been reported^17–19^.

**Figure 2.**
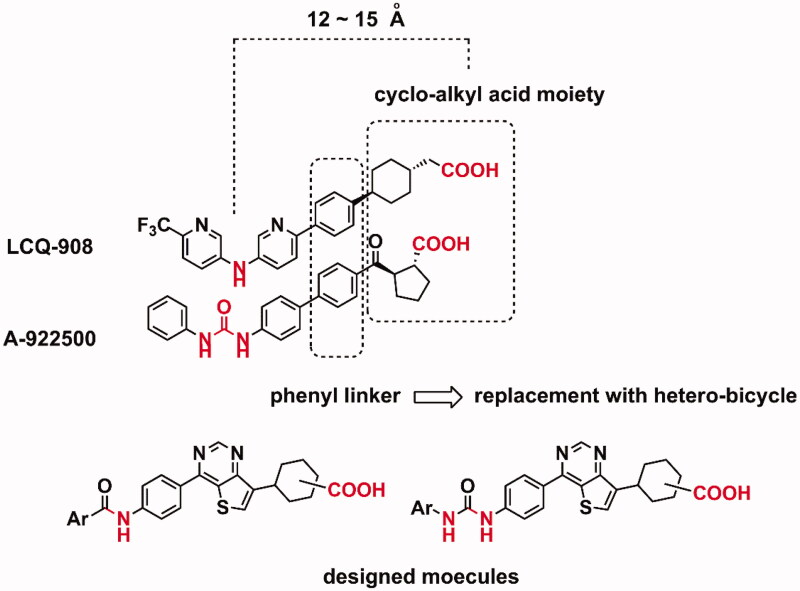
A schematic strategy for the design of target molecules.

Herein, we described the discovery of novel thieno[3,2-*d*]pyrimidine derivatives as DGAT-1 inhibitors. Target molecules were designed based on a bioisosteric replacement strategy (Figure 2). Introduction of a heterobicylcle instead of a phenyl linker was attempted. Thienopyrimidines showed various pharmacologic activities as a privileged scaffold^20^, and the introduction of thieno[3,2-*d*]pyrimidine was the first approach to designing novel inhibitors based on the structural features of reported DGAT-1 inhibitors.

## Materials and methods

### Chemistry

All commercial chemicals and solvents were reagent grade and were used without further purification. Completion of the reactions was monitored by analytical thin layer chromatography (TLC) using precoated glass-backed plates (E-Merck, silica gel 60 F_254_ 0.25 mm). For normal pressure and flash column chromatography purifications, Merck silica gel 60 (size 70–230 and 230–400 mesh, respectively) was used. ^1^H and 13C NMR spectra were recorded with an Avance DPX-300 NMR spectrometer (Bruker, Germany) and Jeol 600 MHz spectrometer (Jeol Resonance ECZ 600 R, USA). All the ^1^H and ^13^C NMR spectra were recorded in deuterated chloroform (CDCl_3_) with tetramethylsilane (TMS) as an internal standard or deuterated dimethyl sulfoxide (DMSO)-*d6* as solvents; chemical shifts are reported in *δ* values (ppm) relative to the residual solvent peak. Multiplicity was indicated as follows: s (singlet); d (doublet); t (triplet); m (multiplet); dd (doublet of doublet); brs (broad singlet). Mass spectra were obtained on Waters Aquity UPLC/QTOF (Waters Corporation, USA). HPLC was used Agilent, 1200 series using capcellpak MGII (4.6 × 150 mm, 5 μm) eluted with a 30 min gradient from 20–70% acetonitrile in water.

### Synthesis of 17a and 17b

#### *Tert*-butyl (4–(7-bromothieno[3,2-d]pyrimidin-4-yl)phenyl)carbamate (8)

A mixture of 7-bromo-4-chlorothieno[3,2-*d*]pyrimidine **6** (13 g, 52.10 mmol, CY C37043, KindChem Corporation, China), *t*-butyl 4–(4,4,5,5-tetramethyl-1,3,2-dioxaborolan-2-yl) phenylcarbamate **7** (20 g, 62.70 mmol**)**, tetrakis(triphenylphosphine)palladium (3.16 g, 2.73 mmol) and sodium carbonate (8.28 g. 78.12 mmol) were added to 100 mL of 1,4-dioxane/water (4:1), and then stirred at 80 °C for 18 h under argon atmosphere. The organic layer was extracted with 300 mL of ethyl acetate and 300 mL of water, dried over anhydrous magnesium sulphate, and then concentrated *in vacuo*. Subsequently, 30 mL of methanol was added, and the resulting mixture was stirred to precipitate out solids, and then filtered to obtain 12.5 g of the yellow title compound.

^1^H-NMR (300 MHz, CDCl_3_): *δ* 9.80(s, 1H), 8.18(d, *J* = 6.9 Hz, 2H), 8.04(s, 1H), 7.62(d, *J* = 6.9 Hz, 2H), 6.76(s, 1H), 1.58(s, 9H).

#### Methyl 2–(4–(4-(4-((*tert*-butoxycarbonyl)amino)phenyl)thieno[3,2-d]pyrimidin-7-yl)cyclohex-3-en-1-yl)acetate (10)

A mixture of **8 (**0.56 g, 1.38 mmol), 2–(4-(4,4,5,5-tetramethyl-1,3,2-dioxaborolan-2-yl)cyclohex-3-en-1-yl)acetate **9** (0.5 g, 1.78 mmol), which was prepared according to the reported method^21^, and tetrakis(triphenylphosphine)palladium (94.9 mg, 0.08 mmol) was added to a solution of 2.06 mL of 2 *N* aqueous sodium carbonate and 8.2 mL of 1,4-dioxane, and then stirred at 100 °C for 12 h under argon. The reaction mixture was extracted with 300 mL of ethyl acetate and 300 mL of water. The organic layer was dried with anhydrous magnesium sulphate, filtered and then concentrated. Subsequently, methanol was added to the resulting solution, stirred to precipitate out solids, and then filtered to obtain 315 mg of the yellow title compound.

^1^H-NMR (300 MHz, CDCl_3_): *δ* 9.30(s, 1H), 8.17(d, *J* = 8.7 Hz, 2H), 7.43(s, 1H), 7.59(d, *J* = 8.7 Hz, 2H), 7.16(bs, 1H), 6.69(s, 1H), 3.71(s, 3H), 2.64 ∼ 2.38(m, 3H), 2.2 6 ∼ 2.02(m, 6H), 1.56(s, 9H). LCMS (ESI) m/z 480.2 [M + H]^+^; HRMS calcd for C_26_H_29_N_3_O_4_S [M + H]^+^ 480.1957, found 480.1986

#### Methyl 2–(4–(4-(4-((t-butoxycarbonyl)amino)phenyl)thieno[3,2-*d*]pyrimidin-7-yl)cyclohexyl) acetate (11)

To a solution of **10** (315 mg, 0.66 mmol) in ethanol (50 mL) and 1,4-dioxane (20 mL) was added 20% charcoal-shaped palladium hydroxide (158 mg, 50%w/w). The mixture was stirred at room temperature under H_2_ overnight. The reaction mixture was filtered through Celite, and then the filtrate was concentrated to obtain the title compound **11** (190 mg), which was used in the following step without further purification.

^1^H-NMR (300 MHz, CDCl_3_): *δ* 9.26(s, 2H), 8.17(d, *J* = 8.5 Hz, 4H), 7.69(s, 1H), 7.63(s, 1H), 7.62(d, *J* = 8.2 Hz, 4H), 6.71(s, 2H), 3.69(s, 6H), 3.3 2 ∼ 3.17(m, 2H), 2.50(d, *J* = 7.5 Hz, 2H), 2.4 3 ∼ 2.35(m, 1H), 2.33(d, *J* = 6.6 Hz, 2H), 2.2 8 ∼ 2.18(m, 2H), 2.0 7 ∼ 1.19(m, 9H), 1.7 3 ∼ 1.60(m, 4H), 1.57(s, 18H), 1.4 0 ∼ 1.25(m, 2H). LCMS (ESI) m/z 482.2 [M + H]^+^; HRMS calcd for C_26_H_31_N_3_O_4_S [M + H]^+^ 482.2114, found 482.2258

#### Methyl 2–(4–(4-(4-aminophenyl)thieno[3,2-*d*]pyrimidin-7-yl)cyclohexyl)acetate (12)

To a solution of **11** (280 mg, 0.58 mmol) in dichloromethane (5 mL) was added 0.6 mL of trifluoroacetic acid. The mixture was stirred at room temperature for 12 h. Then the reaction mixture was concentrated to a residue that was partitioned between dichloromethane and saturated aqueous sodium bicarbonate. The organic layer was separated from the aqueous layer, washed with brine, dried over anhydrous magnesium sulphate, and then concentrated to afford 190 mg of the title compound.

^1^H-NMR (300 MHz, DMSO-*d_6_*): *δ* 9.08(s, 2H), 8.13(s, 1H), 8.04(s, 1H), 8.01(d, *J* = 8.6 Hz, 4H), 6.76(d, *J* = 8.6 Hz, 4H), 5.90(s, 4H), 3.60(s, 6H), 3.3 0 ∼ 3.10(m, 2H), 2.48(d, 2H), 2.28(d, *J* = 6.5 Hz, 2H), 2.2 5 ∼ 2.13(m, 1H), 2.2 8 ∼ 2.16(m, 3H), 2.1 0 ∼ 1.98(m, 2H), 1.9 0 ∼ 1.46(m, 14H), 1.3 0 ∼ 1.10(m, 2H).

#### Methyl 2–(4–(4-(4-(3–(3-chlorophenyl)ureido)phenyl)thieno[3,2-d]pyrimidin-7-yl)cyclohexyl)acetate (13)

To a solution of **12** (3.1 g, 8.13 mmol) in anhydrous tetrahydrofuran (60 mL) was added 3-chlorophenyl isocyanate (1.37 g, 8.92 mmol). The mixture was stirred at room temperature for 18 h. The mixture was concentrated in part and diluted with diethyl ether to give a precipitate, which was filtered to afford 4.06 g of a light-yellow title compound.

^1^H-NMR (300 MHz, DMSO-*d_6_*): *δ* 9.21(s, 2H), 9.16(s, 2H), 9.03(s, 2H), 8.24(s, 1H), 8.2 1 ∼ 8.15(m, 5H), 7.7 4 ∼ 7.71(m, 6H), 7.4 0 ∼ 7.29(m, 4H), 7.1 0 ∼ 7.00(m, 2H), 3.61(s, 6H), 3.3 2 ∼ 3.04(m, 2H), 2.46(d, 2H), 2.29(d, *J* = 6.5 Hz, 2H), 2.2 2 ∼ 2.14(m, 1H), 2.2 1 ∼ 2.01(m, 2H), 1.8 9 ∼ 1.49(m, 14H), 1.3 0 ∼ 1.11(m, 2H). LCMS (ESI) m/z 535.2 [M + H]^+^; HRMS calcd for C_28_H_27_ClN_4_O_3_S [M + H]^+^ 535.1571, found 535.1793

#### Methyl *cis*-2–(4–(4-(4-(3–(3-chlorophenyl)ureido)phenyl)thieno[3,2-d]pyrimidin-7-yl)cyclohexyl)acetate (15a)

**13** (150 mg, 0.28 mmol) was added to ethyl acetate (2 mL) and stirred at 70 °C for 12 h, and then cooled to room temperature to give a solid. The solid (**15b**, 72 mg) was filtered, and then the filtrate was concentrated and the residue was purified by silica gel column chromatography to give 76 mg of the yellow title compound **15a**.

^1^H-NMR (300 MHz, DMSO-*d_6_*): *δ* 9.23(s, 1H), 9.17(s, 1H), 9.04(s, 1H), 8.25(s, 1H), 8.19(d, *J* = 8.8 Hz, 2H), 7.7 5 ∼ 7.72(m, 3H), 7.3 3 ∼ 7.31(m, 2H), 7.0 7 ∼ 7.03(m, 1H), 3.61(s, 3H), 3.3 1 ∼ 3.18(m, 1H), 2.52(d, 2H), 2.2 8 ∼ 2.14(m, 1H), 1.8 9 ∼ 1.49(m, 8H). LCMS (ESI) m/z 535.2 [M + H]^+^; HRMS calcd for C_28_H_27_ClN_4_O_3_S [M + H]^+^ 535.1571, found 535.1793

#### Methyl *trans*-2–(4–(4-(4-(3–(3-chlorophenyl)ureido)phenyl)thieno[3,2-d]pyrimidin-7-yl)cyclohexyl)acetate (15b)

^1^H-NMR (300 MHz, DMSO-*d*_6_): *δ* 9.23(s, 1H), 9.23(s, 1H), 9.16(s, 1H), 9.03(s, 1H), 8.19(d, *J* = 8.8 Hz, 2H), 8.16(s, 1H), 7.7 5 ∼ 7.72(m, 3H), 7.3 6 ∼ 7.29(m, 2H), 7.0 7 ∼ 7.03(m, 1H), 3.61(s, 3H), 3.11(t, *J* = 11.9 Hz, 1H), 2.30(d, *J* = 6.5 Hz, 2H), 2.1 3 ∼ 2.01(m, 2H), 1.9 0 ∼ 1.72(m, 3H), 1.6 8 ∼ 1.50(m, 2H), 1.2 9 ∼ 1.11(m, 2H). LCMS (ESI) m/z 535.2 [M + H]^+^; HRMS calcd for C_28_H_27_ClN_4_O_3_S [M + H]^+^ 535.1571, found 535.1793

#### *cis*-2–(4–(4-(4-(3–(3-chlorophenyl)ureido)phenyl)thieno[3,2-d]pyrimidin-7-yl)cyclohexyl)acetic acid (16a)

To a solution of **15a** (7.8 g, 14.58 mmol) in tetrahydrofuran/methanol/water (120 mL, 1:1:1) was added 1.17 g of sodium hydroxide (29.25 mmol), and the mixture was stirred. The reaction mixture was acidified with 1 *N* HCl (pH was adjusted to between 5 and 6) to give a solid. The solid was filtered, and washed with water to afford **16a** quantitatively.

^1^H-NMR (300 MHz, DMSO-*d_6_*): *δ* 9.28(s, 1H), 9.23(s, 1H), 9.16(s, 1H), 8.24(s, 1H), 8.19(d, *J* = 8.8 Hz, 2H), 7.7 5 ∼ 7.72(m, 3H), 7.3 3 ∼ 7.31(m, 2H), 7.1 0 ∼ 7.00(m, 1H), 3.3 2 ∼ 3.20(m, 1H), 2.39(d, *J* = 7.5 Hz, 2H), 2.2 8 ∼ 2.14(m, 1H), 1.9 0 ∼ 1.50(m, 8H). ^13^C -NMR (150 MHz, DMSO-*d*_6_): *δ* 174.08, 160.01, 158.32, 154.04, 152.20, 142.37, 142.10, 141.00, 133.20, 131.36, 130.43, 129.97, 129.20, 127.20, 121.70, 118.21, 117.66, 116.75, 37.03, 35.51, 30.03, 29.31, 27.18. LCMS (ESI) m/z 521.1 [M + H]^+^; HRMS calcd for C_27_H_25_ClN_4_O_3_S [M + H]^+^ 521.1414, found 521.1441

#### *trans*-2–(4–(4-(4-(3–(3-chlorophenyl)ureido)phenyl)thieno[3,2-d]pyrimidin-7-yl)cyclohexyl)acetic acid (16b)

##### 16b was obtained from the procedure described for 16a

^1^H-NMR (300 MHz, DMSO-*d*_6_): *δ* 12.04(s, 1H), 9.29(s, 1H), 9.23(s, 1H), 9.16(s, 1H), 8.18(d, *J* = 9.0 Hz, 2H), 8.15(s, 1H), 7.7 5 ∼ 7.72(m, 3H), 7.3 6 ∼ 7.29(m, 2H), 7.0 7 ∼ 7.02(m, 1H), 3.11(t, *J* = 12.1, 1H), 2.19(d, *J* = 6.7 Hz, 2H), 2.1 3 ∼ 2.01(m, 2H), 1.9 0 ∼ 1.72(m, 3H), 1.6 8 ∼ 1.50(m, 2H), 1.2 9 ∼ 1.11(m, 2H). ^13^C -NMR (150 MHz, DMSO-*d*_6_): *δ* 173.90, 159.93, 158.35, 154.10, 152.22, 142.36, 141.05, 133.19, 130.81, 130.41, 129.97, 129.17, 127.27, 121.68, 118.25, 117.70, 116.79, 41.60, 36.15, 34.13, 32.42, 32.05. LCMS (ESI) m/z 521.1 [M + H]^+^; HRMS calcd for C_27_H_25_ClN_4_O_3_S [M + H]^+^ 521.1414, found 521.1441

#### Sodium *cis*-2–(4–(4-(4-(3–(3-chlorophenyl)ureido)phenyl)thieno[3,2-d]pyrimidin-7-yl)cyclohexyl)acetate (17a)

To a solution of **16a** (7.6 g, 14.59 mmol) in 100 mL of methanol was added 14 mL of a 1 *N* sodium hydroxide. The resulting mixture was stirred at room temperature for 2 h. The solvent was removed to give 8.3 g of the yellow title compound **17a.**

^1^H-NMR (300 MHz, DMSO-*d*_6_): *δ* 12.50(s, 1H), 12.37(s, 1H), 9.21(s, 1H), 8.23(s, 1H), 8.17(d, *J* = 8.8 Hz, 2H), 7.7 5 ∼ 7.72(m, 3H), 7.56 (d, *J* = 9.0 Hz, 1H), 7.27 (t, *J* = 8.1 Hz, 1H), 6.96 (d, *J* = 7.9 Hz, 1H), 3.3 2 ∼ 3.18(m, 1H), 2.4 0 ∼ 2.20(m, 3H), 2.0 0 ∼ 1.60(m, 8H). ^13^C -NMR (150 MHz, DMSO-*d*_6_): *δ* 178.28, 160.00, 158.50, 154.04, 153.53, 144.38, 142.88, 142.70, 132.90, 130.91, 130.04, 128.93, 128.77, 126.97, 120.44, 117.95, 117.40, 116.55, 41.40, 39.08, 30.92, 30.03, 27.70. LCMS (ESI) m/z 521.1 [M + H]^+^; HRMS calcd for C_27_H_25_ClN_4_O_3_S [M + H]^+^ 521.1414, found 521.1441 (as free base)

#### Sodium *trans*-2–(4–(4-(4-(3–(3-chlorophenyl)ureido)phenyl)thieno[3,2-d]pyrimidin-7-yl)cyclohexyl)acetate (17b)

^1^H-NMR (300 MHz, DMSO-*d*_6_): *δ* 12.27(s, 1H), 12.25(s, 1H), 9.18(s, 1H), 8.17(d, *J* = 8.9 Hz, 2H), 7.99(s, 1H), 7.89(d, *J* = 8.9 Hz, 2H), 7.83(s, 1H), 7.56(d, *J* = 9.3 Hz, 1H), 7.27(t, *J* = 8.1 Hz, 1H), 6.96(d, *J* = 8.0 Hz, 1H), 3.11(t, *J* = 11.6, 1H), 2.1 0 ∼ 1.83(m, 2H), 1.6 8 ∼ 1.46(m, 2H), 1.2 9 ∼ 1.11(m, 2H). ^13^C -NMR (150 MHz, DMSO-*d*_6_): *δ* 178.21, 159.84, 158.38, 154.07, 153.42, 144.40, 142.93, 142.83, 132.90, 130.28, 130.05, 128.94, 128.60, 126.87, 120.48, 117.80, 117.32, 116.49, 46.16, 36.07, 35.92, 33.08, 32.68. LCMS (ESI) m/z 521.1 [M + H]^+^; HRMS calcd for C_27_H_25_ClN_4_O_3_S [M + H]^+^ 521.1414, found 521.1441 (as free base)

### DGAT-1 inhibition assay (IC_50_)

The activity of DGAT-1 inhibitors *in vitro* was evaluated by using a human recombinant DGAT1 enzyme expressed in insect cells (SF9 cells). SF9 cells were homogenised by washing them with DPBS (Dulbecco’s phosphate-buffered saline) and then suspending cell pellets with a tris buffer (250 mM sucrose; 10 mM Tris-HCl [pH 7.4]; proteinase inhibitor). The resulting mixture was centrifugally separated at 10,000 × *g* for 30 min to remove the cell debris remaining in the lower layer thereof, and was centrifugally separated at 100,000 × *g* for 60 min to obtain a microsomal membrane. Further, membrane fractions were resuspended by the tris buffer, and then stored at -80 °C.

The activity of DGAT1 was measured according to the reported method^20^. Specifically, 0.0001 – 10 μΜ (final concentration, FC) of the test compounds were cultured at room temperature (25 °C) for 15 min with a 10 μΜ of SF9 microsomal protein solution and 100 mM of MgCl_2_ solution, and were then further cultured at room temperature (25 °C) for 30 min after the addition of 100 μΜ (FC in 12.5% EtOH) of 1,2-dioleyl-sn-glycerol and 30 μΜ (final conc.) of ^14^C-oleyl coenzyme A. The reaction was completed by the addition of 300 μL of a mixed solution of 2-propanol/heptane (7:1), and radioactive triglyceride was separated from an organic solvent layer by using 200 μL of heptane and 200 μL of a 0.1 M carbonate buffer (pH 9.5). The amount of triglyceride was measured by liquid scintillography (Perkin Elmer, USA) after mixing with an organic solvent and an equivalent amount of scintillation solvent (MicroScint-O, Perkin Elmer, USA). The effects of inhibiting DGAT1 were calculated as percent in comparison with a control material.

### Oral lipid tolerance test (OLTT)

#### Mouse

Male, Institute of Cancer Research (ICR) mice were obtained from OrientBio Inc. (Republic of Korea). ICR mice (8-weeks old, *n* = 5/group) fasted 16 h, received 5 mL/kg corn oil via oral administration (p.o.) 30 min after compound administration. Plasma was collected at different time points (0, 1, 2, and 4 h).

#### Dog

Beagle dogs (9 months old, *n* = 2/group) were obtained from Woojung Bio Inc. (Republic of Korea) were fasted for 16 h and received heavy cream (2.7 mL/kg), glucose (1 g/kg), water (5.3 mL/kg), and acetaminophen (20 mg/kg p.o.) 1 h after compound administration. Blood samples were collected at –0.5, 0, 1, 2, and 4 h.

### Statistical analysis

Statistical analysis was performed using one-way ANOVA, followed by Dunnett’s multiple comparison test using Prism 6.0 (GraphPad, USA). The Kruskal-Wallis test was applied when data did not pass the normality test.

## Results and discussion

**Scheme 1.**
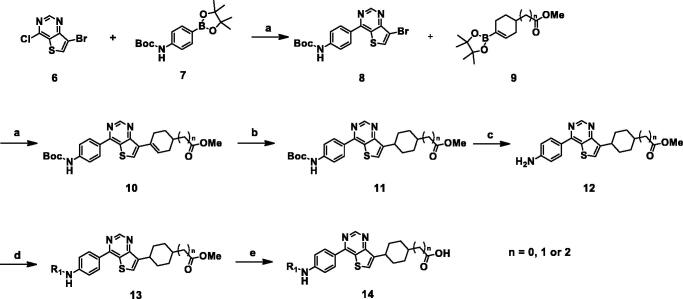
Synthesis of thienopyrimidine derivatives. Reagents and conditions: (a) Pd(PPh_3_)_4_, 2 N Na_2_CO_3_, 1,4-Dioxane; (b) H_2_, 20% PdOH, EtOH/1,4-Dioxane; (c) TFA, DCM, rt; (d) aryl chloride, pyridine, DCM 0 °C to rt or aryl isocyanate, THF, 12 h, rt; (e) NaOH or LiOH, THF/MeOH/H_2_O (1:1:1), rt.

**Scheme 2.**
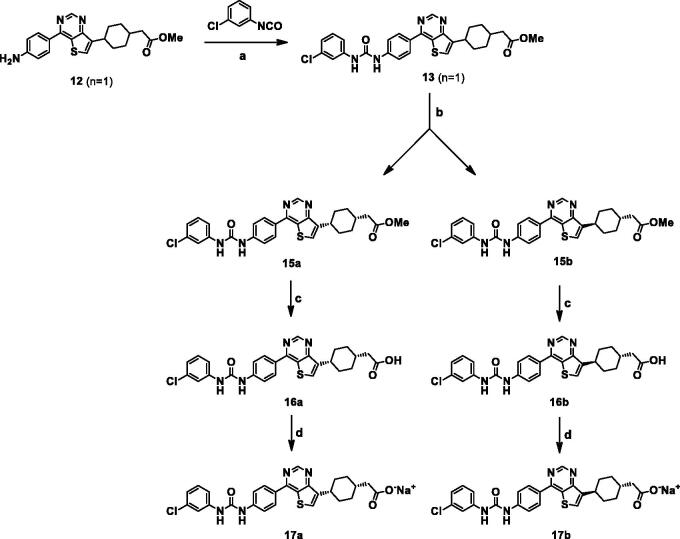
Reagents and conditions: (a) THF, rt; (b) separation by using ethyl acetate at 60–70 °C; filtrate for **15a** and solid for **15b**; (c) NaOH, THF/MeOH/H_2_O; (d) 1 M NaOH, MeOH, rt.

We found that thieno[3,2-*d*]pyrimidine could be a core scaffold connecting an aryl moiety at one end to a cyclo-aliphatic carboxylic acid group at the other. Synthetic schemes for a series of thieno[3,2-*d*]pyrimidine derivatives are outlined in Schemes 1 and 2.

Desired compounds **14a**–**14l** were synthesised from commercially available 7-bromo-4-chlorothieno[3,2-*d*]pyrimidine (**6)**, as shown in Scheme 1. Consecutive Suzuki coupling reactions gave **8** and **10.** Compound **9** was prepared using the reported method^19^. Hydrogenation of **10**, followed by de-protection of the Boc group with trifluoroacetic acid afforded the key intermediate **12**. The aniline **12** was converted to amides **14a**–**14h** or ureas **14i**–**14l** by coupling with acid chlorides or aryl isocyanates, respectively, followed by hydrolysis with sodium hydroxide or lithium hydroxide.

The activities of synthesised compounds were evaluated using SF9 insect cells expressing human DGAT-1^22^. The IC_50_ values are summarised in Table 1. A potent DGAT-1 inhibitor, **LCQ-908** (**2** or pradigastat) was used as a positive control. To optimise the alkyl chain length of the acid moiety, compound **14b** (*n* = 1) was more potent than short chain **14a** (*n* = 0) and long chain **14c** (*n* = 2). The calculated lowest energy conformation model of **14c** provided that the distance between the terminal carboxylic acid and NH of urea was over 16 Å, which may result in reduced activity. **14a** exhibited a loss of activity, which suggested that the terminal carboxylic acid should be placed one-carbon away from cyclohexane. Next, structure-activity relationships for the aryl moiety on the left side were investigated, with derivatives bearing chain lengths the same as that of **14b**. 5-chloro-2-nitro phenyl (**14d)**, 2-trifluoromethylpyridinyl (**14f)**, 4-chloropyridinyl (**14k)**, and 5-bromopyridinyl (**14l)** derivatives had no activity (IC_50_ >1 μM), while **14b**, **14c**, **14e**, **14h**, and **14j** had good activity (IC_50_ values of 0.3 μM–0.4 μM). Compounds **14g** and **14i** were equipotent to the known compound **2**. The most potent compound, **14i**, was subjected to further investigation. *Cis* and *trans* isomers of **14i** were separated to determine the activity and properties of each isomer. As shown in Scheme 2, the racemic mixture **13** was conveniently separated into *cis*-isomer **15a** and *trans*-isomers **15b** by the treatment with ethyl acetate. **15a** and **15b** exhibited greatly different solubility for ethyl acetate, and were soluble and insoluble for ethyl acetate, respectively. Hydrolysis of **15a** and **15b** afforded *cis* isomer **17a** and *trans* isomer **17b,** respectively.

**Table 1.** DGAT-1 inhibitors and their *in vitro* data.

^a^ Data present mean values.

Interestingly, the isomers **17a** and **17b** were more potent than mixture **14i**, and even compound **2**, in SF9 cells (Table 2). However, pharmacokinetic studies demonstrated that *cis*-isomer **17a** had a much better profile than *trans*-isomer **17b** (Table 3). **17a** had a shorter half-life (1.2 h), but had much better bioavailability, compared to **17b**. In enzymatic assays, **17a** had an IC_50_ of 61 nM for DGAT-1 and displayed high off-target selectivity against DGAT-2, acyl-coenzyme A (CoA):cholesterol acyltransferases (ACAT1 and ACAT2) (Table 2). ACATs have sequence homology to DGAT-1 and play essential roles in cholesterol homeostasis^23^. **17b** was not explored in the enzymatic assays due to its poor pharmacokinetic profile.

**Table 2.** Enzyme and cellular inhibitory potencies for selected compounds.

IC_50_ (nM)					
Compound	DGAT1(SF9)	hDGAT1	hDGAT2	hACAT1	hACAT2
**2**	157	57	>10,000	>10,000	>10,000
**17a**	74	61	>10,000	>10,000	>10,000
**17b**	89	–	–	–	–

**Table 3.** Pharmacokinetic profiles of compounds **17a, 17b** in ICR mice.^a^

Compounds	17a	17b
AUC _0∼24_ (ng·h/mL)	861.2	233.7
*C*_max_ (ng/mL)	479.3	74.8
T_1/2_ (h)	1.2	5.2
Bioavailability (%)	19	0.7

^a^ All parameters were determined after oral administration (10 mg/kg, *n* = 3) and intravenous (iv) injection (3 mg/kg, *n* = 2) in ICR mice; both compounds were dissolved in a vehicle solution of DMSO/Tween80/saline (1:6:23) for iv., and 0.5% methylcellulose/0.5% Tween80 in distilled water for oral administration. Data represent mean values.

Considering potency, selectivity, and pharmacokinetic profiles, **17a** was investigated for further pharmacokinetic properties in four species, and results are summarised in Tables 4 and 5. **17a** exhibited low to moderate clearance and acceptable oral bioavailability in rat and dog.

**Table 4.** Oral pharmacokinetic profile of **17a** in rat and dog.

Parameters	AUC 0 ∼ 24 (ng·h/mL)	*T* 1/2 (h)	Cl (L/hr/kg))	Bioavailability (%)
Rat^a^	18,594.5 ± 6764.2	3.0 ± 0.4	0.5 ± 0.2	30
Dog^b^	8243.0 ± 2296.3	3.6 ± 0.7	0.4 ± 0.1	53

^a^ Dose; 3 mg/kg for iv, 10 mg/kg for po.; Vehicle: DMSO/Tween80/saline (1:6:23) for iv.; 0.5% methylcellulose/0.5% Twee80 in distilled water for po.

^b^ Dose: 1 mg/kg for iv, 3 mg/kg for po.; Vehicle: DMSO/Tween80/saline (1:3:26) for iv; 0.5% methylcellulose/0.5% Tween80 in distilled water for po.

**Table 5.** *In vitro* microsomal stability and cytochrome P450 (CYP) inhibition assay of compound **17a.**

	Microsomal stability, (%)^a^				CYPs enzyme inhibition, IC_50_ (μM)^b^				
No	Human	Dog	Rat	Mouse	CYP3A4	CYP1A2	CYP2C9	CYP2C19	CYP2D6
**17a**	94	82	87	82	16.2	10.6	2.9	>20	8.7

^a^ Remaining percent of metabolism by incubation of the parent molecule (5 μM) with liver microsomes of mouse, rat, dog, and human for 60 min (duplicate).

^b^ Data were analysed by a fluorescence detection method.

**17a** exhibited durable metabolic stabilities, as shown in Table 5. In particular, **17a** was metabolically stable in four species: human, dog, rat, and mouse. Further evaluation of **17a** was carried out for inhibition of clinically relevant cytochrome 450 (CYP) isoforms (3A4, 1A2, 2C9, 2C19, 2D6). No significant inhibition by **17a** was observed (Table 5). Thus, **17a** would be very unlikely to affect the pharmacokinetics of other drugs.

**Figure 3.**
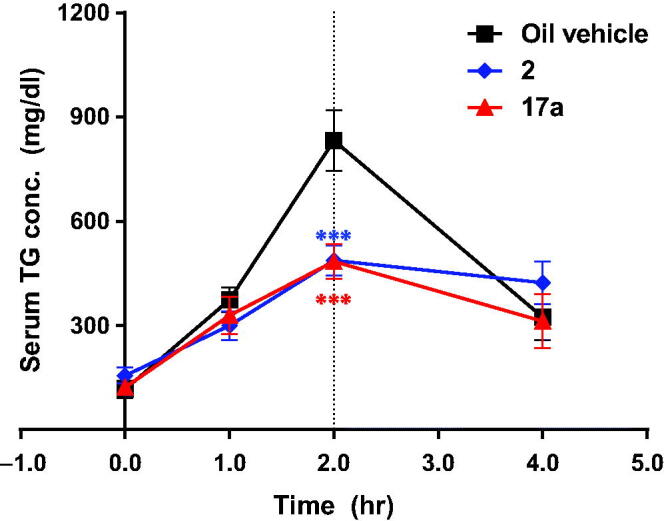
Effect of **17a** in the oral lipid tolerance test (*n* = 5, ***p < .001). Serum TG (or TAG) concentration was measured before and 1, 2, and 4 h after oral administration of corn oil in ICR mice treated with **17a** (3 mg/kg) or **2** (1 mg/kg). Vehicle for **2**: 0.5% methylcellulose/0.5% Tween80 in distilled water, vehicle for **17a**: 0.5% Tween80 in distilled water.

**Figure 4.**
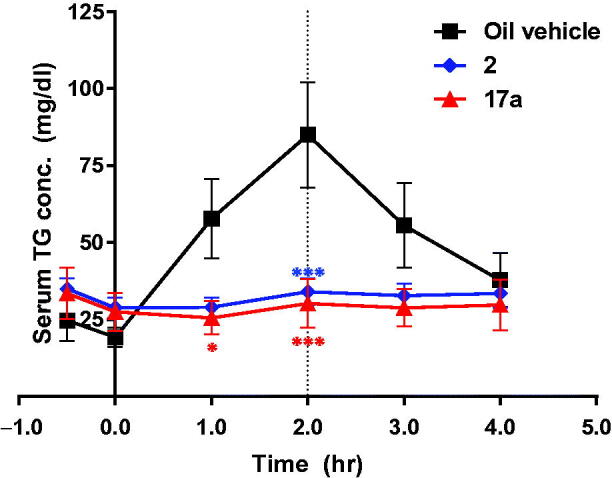
Effect of **17a** in a canine oral lipid tolerance test (*n* = 2, *p < .05; ***p < .001). The dog was treated with **17a** (po. 1 mg/kg) or **2** (po. 1 mg/kg) before oral administration of corn oil. Vehicle for **2**: 0.5% methylcellulose/0.5% Tween80 in distilled water, vehicle for **17a**: 0.5% Tween80 in distilled water.

To determine *in vivo* efficacy, we evaluated the activity of **17a** against serum TAG level induced by the oral administration of corn oil (oral lipid tolerance test, OLTT; Figures 3 and 4), since intestinal targeted DGAT-1 inhibition results in a reduced serum TAG level^14^. **17a** was orally administered to ICR mice at 3 mg/kg or to dogs at 1 mg/kg before administration of corn oil. As shown in Figure 3, **17a** showed significant activity, as potent as compound **2,** in reducing plasma TAG level.

**Figure 5.**
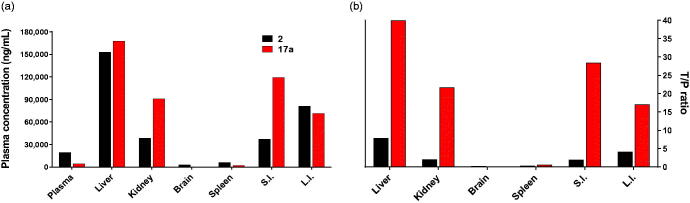
Tissue distribution of **17a** in mice. **17a** and **2** were orally administered to ICR mice at 30 mg/kg (*n* = 3). (a) AUC values of **17a** and **2** in indicated tissues. Each bar shows AUC obtained from concentrations measured at 0.5 h, 1 h, 2 h, 4 h, and 24 h after dosing. Data represent mean value. (b) Tissue-to-plasma (T/P) ratio of **17a** and **2**. S.I.: small intestine, L.I.: large intestine.

In addition, tissue distribution of **17a** was investigated. **17a** was orally administered to mice at 30 mg/kg, and **2** was administrated as a positive control (Figure 5). The concentration levels of **17a** in the liver and small intestine were much higher than in other tissues, indicating that liver and small intestine are the main target organs of **17a**, while **2** showed much high concentrations in the liver and large intestine. Analysis of tissue-to-plasma ratios indicated that **17a** had favourable distributions in the liver and small intestine. Significant activity of **17a** in the OLTT appeared to result from high concentration in the small intestine, even though bioavailability of **17a** was not great in mice. This result could be proof of a pharmacokinetic–pharmacodynamic correlation.

## Conclusion

We found that a novel series of thieno[3,2-*d*]pyrimidine derivatives had potent DGAT-1 inhibitory activity. A representative compound, *cis*-isomer **17a** showed potent DGAT-1 inhibitory activity, metabolic stability in four species, an acceptable pharmacokinetic profile in rodents and dogs, and a significant reduction of TAG in the OLTT in mice and dogs. In a tissue-distribution study, **17a** was mainly distributed to the liver and small intestine. Therefore, **17a** could be a candidate with considerable potential and deserves for further investigation.
